# CDDO-Me Attenuates CA1 Neuronal Death by Facilitating RalBP1-Mediated Mitochondrial Fission and 4-HNE Efflux in the Rat Hippocampus Following Status Epilepticus

**DOI:** 10.3390/antiox11050985

**Published:** 2022-05-18

**Authors:** Ji-Eun Kim, Duk-Shin Lee, Tae-Hyun Kim, Tae-Cheon Kang

**Affiliations:** Department of Anatomy and Neurobiology and Institute of Epilepsy Research, College of Medicine, Hallym University, Chuncheon 24252, Korea; jieunkim@hallym.ac.kr (J.-E.K.); dslee84@hallym.ac.kr (D.-S.L.); hyun1028@hallym.ac.kr (T.-H.K.)

**Keywords:** FJB, mitochondrial dynamics, mitochondrial elongation, mitochondrial fragmentation, oxidative stress, seizure

## Abstract

Ras-related protein Ral-A (RalA)-binding protein 1 (RalBP1, also known as Ral-interacting protein of 76 kDa (RLIP76) or Ral-interacting protein 1 (RLIP1 or RIP1)) is involved in the efflux of 4-hydroxynonenal (4-HNE, an end product of lipid peroxidation), as well as mitochondrial fission. In the present study, we found that 2-cyano-3,12-dioxo-oleana-1,9(11)-dien-28-oic acid methyl ester (CDDO-Me) attenuated CA1 neuronal death and aberrant mitochondrial elongations in these neurons coupled with enhanced RalBP1 expression and reduced 4-HNE levels following status epilepticus (SE). RalBP1 knockdown did not affect mitochondrial dynamics and CA1 neuronal death under physiological and post-SE conditions. Following SE, however, cotreatment of RalBP1 siRNA diminished the effect of CDDO-Me on 4-HNE levels, mitochondrial hyperfusion in CA1 neurons, and CA1 neuronal death. These findings indicate that CDDO-Me may ameliorate CA1 neuronal death by facilitating RalBP1-mediated 4-HNE efflux and mitochondrial fission following SE. Therefore, our findings suggest that increased RalBP1 expression/activity may be one of the considerable targets to protect neurons from SE.

## 1. Introduction

The morphology of mitochondria is regulated by a balance of fusion (elongation) and fission (fragmentation), which are referred to as mitochondrial dynamics. The maintenance of mitochondrial dynamics is essential for cell development and mitosis. Furthermore, the defects of this process lead to various types of cell death [1,2]. Large GTPase dynamin-related protein 1 (DRP1) is one of the regulators of mitochondrial dynamics, which reversely acts by phosphorylation of distinct sites: phosphorylation of serine (S) 616 site on DRP1 facilitates mitochondrial fission, while S637 leads to fusion by detaching DRP1 from the outer mitochondrial membrane [2,3].

Status epilepticus (SE, uncontrolled prolonged seizure activities) shows a high mortality rate and causes secondary neurological deficits such as neuronal injury, alterations of neuronal networks, brain edema, neuroinflammation, and cognitive impairments [4,5]. In the hippocampus, CA1-3 pyramidal cells and dentate hilar neurons are vulnerable, while dentate granule cells are relatively invulnerable to SE [6,7]. In addition, SE induces distinct types of neuronal death in the heterogeneous patterns of mitochondrial dynamics: SE results in programmed necrosis of CA1 neurons accompanied by impaired mitochondrial fission and apoptosis of hilar parvalbumin (PV) cells (one of the GABAergic interneurons) by excessive mitochondrial fission without affecting optic atrophy 1 and mitofusin expression levels in the pilocarpine-induced SE rat model [2,8,9]. However, the underlying mechanisms of the regulation of mitochondrial dynamics leading to distinct neuronal death patterns have been elusive.

Ras-related protein Ral-A (RalA)-binding protein 1 (RalBP1, also known as Ral-interacting protein of 76 kDa (RLIP76) or Ral-interacting protein 1 (RLIP1 or RIP1)) removes 4-hydroxynonenal (4-HNE, an end product of lipid peroxidation) from the cytoplasm [10]. Thus, impaired RalBP1 activity accumulates 4-HNE, which induces massive apoptosis [11]. Furthermore, RalBP1 fosters DRP1 S616 phosphorylation during mitochondrial fission [3]. Unlike the improper 4-HNE efflux, disruption of RalBP1 leads to aberrant mitochondrial segregation and impaired mitochondrial energy production, which are relevant to programmed necrosis of CA1 neurons [8,12,13,14,15]. However, we have reported that SE leads to the aberrant mitochondrial elongation in CA1 neurons concomitant with the increased RalBP1 expressions. Furthermore, the inhibition of mitochondrial elongation in CA1 neurons attenuates upregulation of RalBP1 expression induced by SE. Therefore, we have speculated that RalBP1 would not be relevant to DRP1-related neuronal death induced by SE and be an adaptive response to SE rather than mitochondrial fission [8].

2-Cyano-3,12-dioxo-oleana-1,9(11)-dien-28-oic acid methyl ester (CDDO-Me) has antioxidant properties, maintaining redox homeostasis as an activator of nuclear factor-erythroid 2-related factor 2 (Nrf2, a redox-sensitive transcription factor) [16]. We have reported that CDDO-Me abolishes upregulation of 4-HNE signals induced by SE. In addition, CDDO-Me activates DRP1-mediated mitochondrial fragmentation, which mitigates CA1 neuronal death induced by SE [17,18]. With respect to the roles of RalBP1 in mitochondrial fission and 4-HNE efflux, it is likely that CDDO-Me may affect SE-induced mitochondrial dynamics and its related CA1 neuronal death by regulating RalBP1 expression, which has been unreported.

Here, we show that CDDO-Me facilitated mitochondrial fissions and attenuated CA1 neuronal death following SE. In addition, CDDO-Me enhanced RalBP1 expression in CA1 neurons concomitant with reduced 4-HNE levels following SE. Although RalBP1 knockdown did not affect mitochondrial dynamics and CA1 neuronal death under physiological and post-SE conditions, cotreatment of RalBP1 siRNA abated reductions in 4-HNE signals, CA1 neuronal degeneration, and mitochondrial elongation induced by CDDO-Me under post-SE conditions. These findings indicate that endogenous RalBP1 may basically participate in 4-HNE efflux but be insufficient to affect mitochondrial dynamics in neurons under physiological and post-SE conditions. At a higher expression level (induced by CDDO-Me), however, RalBP1 may accelerate mitochondrial fission in CA1 neurons following SE. Therefore, our findings suggest that CDDO-Me may protect CA1 neurons from SE insults by facilitating 4-HNE efflux and mitochondrial fission.

## 2. Materials and Methods

### 2.1. Experimental Animals and Chemicals

Male Sprague–Dawley (SD) rats (7 weeks old, *n* = 175, Daehan Biolink, South Korea) were used in the present study. Rats were housed under controlled conditions (22 ± 2 °C, 55 ± 5%, and a 12:12 light/dark cycle with lights) and accessed food and water ad libitum. Four animals died during experimental procedures, and three rats did not respond to pilocarpine treatment, which were ruled out in the present study. Animal experiments were allowed by the Institutional Animal Care and Use Committee of Hallym University (Chuncheon, Korea; No. Hallym 2021-30, approval date: 17 May 2021).

### 2.2. Surgery, CDDO-Me Infusion, and RalBP1 Knockdown

Animals were implanted with Brain Infusion Kit 1 and an Alzet 1007D osmotic pump (Alzet, Cupertino, CA, USA) under isoflurane anesthesia (3% induction, 1.5–2% for surgery, and 1.5% maintenance in a 65:35 mixture of N_2_O/O_2_) and infused with the following chemical or siRNA into the right lateral ventricle (1 mm posterior; 1.5 mm lateral; −3.5 mm depth to the bregma): (1) vehicle, (2) CDDO-Me (10 μM), (3) a nontargeting control siRNA (5-GCAACUAACUUCGUUAGAAUCGUUAUU-3), (4) RalBP1 siRNA (5-CGAAGGAGCUGGAAACCAAtt-3, RNA Accession Number: NM_032067.1), (5) CDDO-Me + control siRNA, and (6) CDDO-Me + RalBP1 siRNA (*n* = 28 in each group). The correct infusion into the lateral ventricle was confirmed during the tissue process for immunohistochemistry and Western blot. A monopolar stainless-steel electrode was also implanted into the left dorsal hippocampus (−3.8 mm posterior; 2.0 mm lateral; −2.6 mm depth) in some animals (*n* = 7 in each group) and secured to the skull with dental acrylic. Three days after surgery, rats were induced with SE by lithium chloride (LiCl)-pilocarpine.

### 2.3. SE Induction and Electroencephalogram (EEG) Analysis

SE was induced by a single dose (30 mg/kg) of pilocarpine in rats pretreated (24 h before pilocarpine injection) with 127 mg/kg LiCl, as previously described [8,9,17,18]. Rats were pretreated with atropine methyl bromide (5 mg/kg i.p.) prior to pilocarpine injection. Two hours after SE, animals were administered diazepam (10 mg/kg, i.p.) to cease behavioral seizures and repeated as needed. As controls, rats were treated with atropine methyl bromide, followed by saline, but not pilocarpine. EEG signals of electrode-implanted animals were recorded with a DAM 80 differential amplifier (0.1–3000 Hz bandpass; World Precision Instruments, Sarasota, FL, USA) during the 2 h recording session for each animal. The EEG signals were digitized and analyzed using LabChart Pro v7 (AD Instruments, New South Wales, Australia). Time of seizure onset was determined when rhythmic paroxysmal depolarizations (4–10 Hz) lasting >3 s with 2 times higher amplitudes than the basal EEG were detected. Two hours after SE onset, diazepam (Valium; Roche, France; 10 mg/kg, i.p.) was administered and repeated as needed.

### 2.4. Tissue Processing

Three days after SE induction, animals (*n* = 7 in each group) were deeply anesthetized with urethane (1.5 g/kg i.p.) and transcardially perfused with phosphate-buffered saline, followed by 4% paraformaldehyde. Brains were excised and then postfixed with the same fixative. Brains were cryoprotected with 30% sucrose and sectioned with a cryostat. For Western blot, the hippocampus was collected after decapitation under urethane anesthesia. After homogenization, protein concentration was determined with a BCA Protein Assay Kit (Pierce Chemical, Rockford, IL, USA).

### 2.5. Immunohistochemistry, Measurements of Mitochondrial Length, and Neuronal Damage

Sections were blocked in 10% normal goat serum (Vector, Burlingame, CA, USA) and subsequently incubated overnight at room temperature in primary antibodies (Table 1). Thereafter, sections were reacted with a fluorescein isothiocyanate- or Cy3-conjugated IgG (Vector, Burlingame, CA, USA) for 1 h. For negative control, preimmune serum was used to substitute for the primary antibody. Fluoro-Jade B (FJB) staining was performed to detect degenerating neurons based on the manufacturer’s instructions [18]. In five hippocampal sections in an animal (five animals in each group), the randomly selected CA1 neurons (total of 25 cells in each group) were used for mitochondrial morphometry using AxioVision Rel. 4.8 and ImageJ software. The parameters for mitochondrial morphometry were as follows: area-weighted form factor (mitochondrial elongation index) = squared perimeter/4π; form factor (the transition of a mitochondrion to elongated and complex morphology) = squared perimeter/4π × area; cumulative area/perimeter ratio (the transition of an elongated mitochondrion to interconnected mitochondria) = Σarea/Σperimeter [19,20]. Fluorescent intensity was measured from 15 sections per animal and then normalized by setting the threshold levels. A blind test was applied to the measurement of mitochondrial length, fluorescent intensity, and cell count of FJB-positive neurons.

### 2.6. Western Blot

Western blot was performed by the standard protocol. Following electrophoresis, proteins were electrotransferred onto nitrocellulose membranes, blocked with 2% bovine serum albumin in Tris-buffered saline, and then incubated with primary antibodies (Table 1) overnight at 4 °C. After washing, membranes were reacted with secondary antibodies. Signals were detected by chemiluminescence using the ECL Western blotting kit (GE Healthcare Korea, Seoul, Korea). The bands were detected and quantified using an ImageQuant LAS4000 system (GE Healthcare Korea, Seoul, Korea) and normalized to β-actin.

### 2.7. Quantification of Data and Statistical Analysis

Data were analyzed by Shapiro–Wilk *W*-test to evaluate the values on normality followed by two-tailed Student’s *t*-test and one-way repeated measure ANOVA. One-way ANOVA, followed by Bonferroni *post hoc* tests, was applied for multiple comparisons. A value of *p* < 0.05 was considered to be statistically significant.

## 3. Results

### 3.1. CDDO-Me Upregulates RalBP1 Protein Levels but Reduces 4-HNE Signals in the CA1 Region Following SE

**Figure 1:**
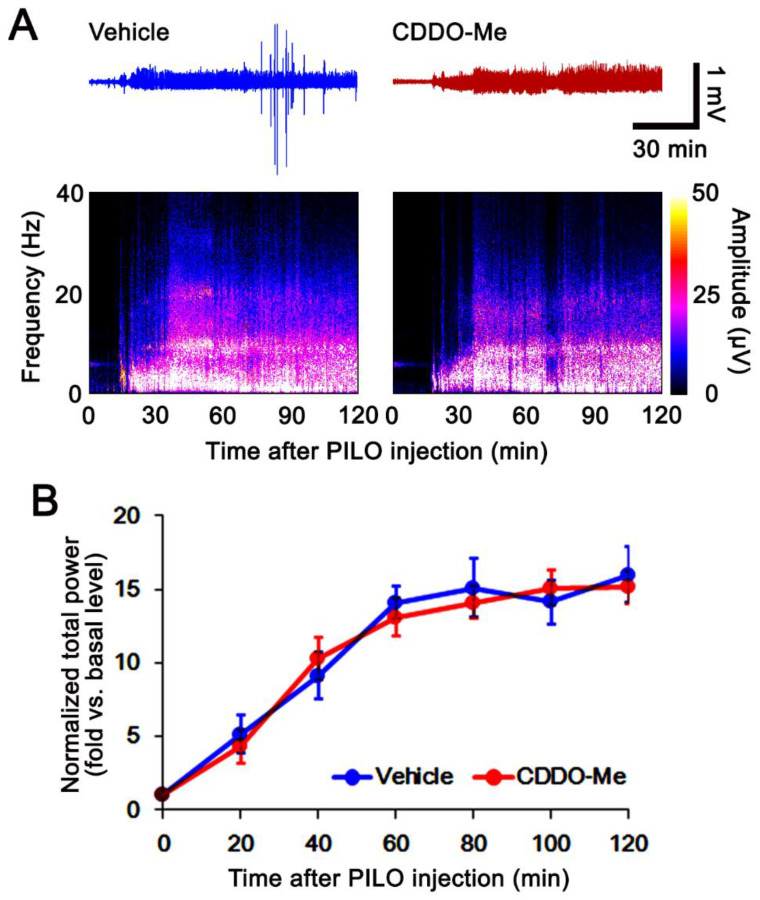
Effects of CDDO-Me on pilocarpine (PILO)-induced seizure activity. CDDO-Me does not alter seizure susceptibility to pilocarpine. (**A**) Representative EEG traces and frequency/amplitude maps following pilocarpine treatment. (**B**) Quantification of the total EEG power (seizure intensity) in response to pilocarpine (mean ± S.E.M.; *n* = 7; repeated-measures ANOVA).

**Figure 2:**
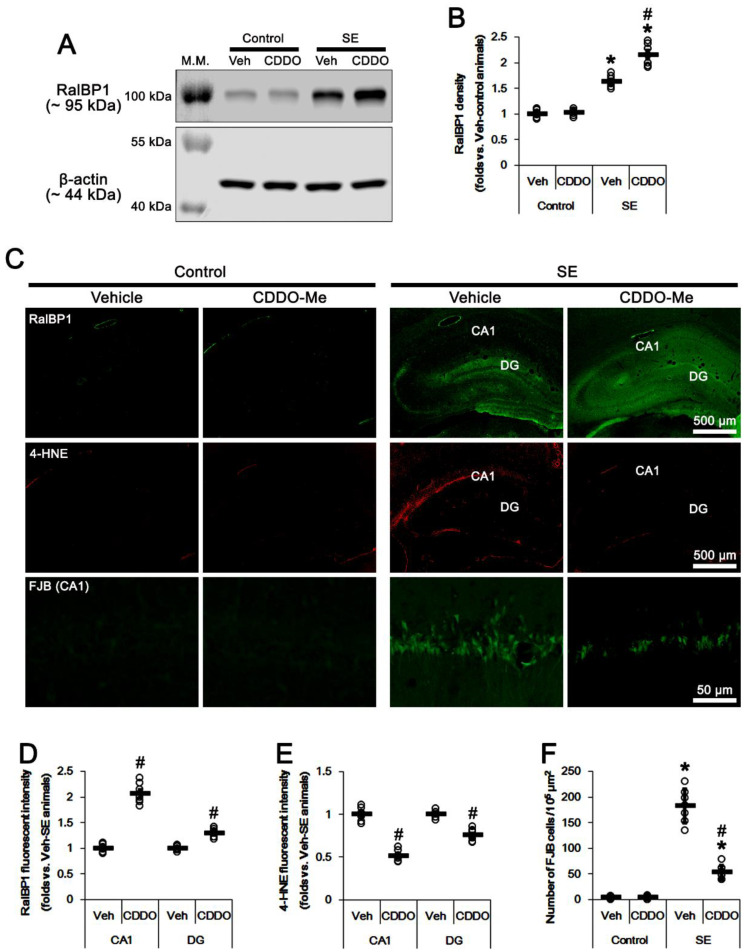
Effects of CDDO-Me on RalBP1 expression, 4-HNE signals, and CA1 neuronal death following SE. As compared to vehicle, CDDO-Me increases RalBP1 protein levels with reduced 4-HEN signals. CDDO-Me also attenuates SE-induced CA1 neuronal death. (**A**) Representative Western blots of RalBP1 protein levels in the whole hippocampus (M.W. marker, molecular weight marker). (**B**) Quantification of RalBP1 protein levels based on Western blot data. (**C**) Representative photos of RalBP1, 4-HNE, and Fluoro-Jade B (FJB) staining images. SE increases RalBP1 levels in the dentate gyrus (DG), but not in the CA1 region, while it induces 4-HNE accumulation in the CA1 region. CDDO-Me ameliorates SE-induced CA1 neuronal death with increased RalBP1 protein levels and reduced 4-HEN signals. (**D**,**E**) Quantification of RalBP1 and 4-HNE fluorescent intensity (mean ± S.E.M.; ^#^ *p* < 0.05 vs. and vehicle; *n* = 7; Student *t*-test). (**F**) Quantification of the number of FJB-positive CA1 neurons following SE (mean ± S.D.; *,^#^ *p* < 0.05 vs. control animals and vehicle; *n* = 7; Student *t*-test). Open circles indicate each individual value. Horizontal bars indicate mean value.

Compatible with previous studies [17,18], the seizure susceptibility to pilocarpine was unaffected by CDDO-Me (repeated one-way ANOVA; *n* = 7; Figure 1). However, SE enhanced RalBP1 protein expression to 1.63-fold of control levels in the whole hippocampus (*t*_(12)_ = 12, *p* < 0.001 vs. vehicle-treated control animals, Student *t*-test, *n* = 7; Figure 2A,B). Immunofluorescent studies demonstrated that RalBP1 expression was rarely or very weakly detected in the hippocampus under physiological conditions. However, SE mainly increased RalBP1 expression in the dentate gyrus, but not the CA1 region (Figure 2C). In contrast to RalBP1, SE induced 4-HNE signals in the CA1 region, but not the dentate gyrus (Figure 2C). Under physiological conditions, CDDO-Me did not influence RalBP1 expression levels in the whole hippocampus (Figure 2A,B and Supplementary Figure S1). However, CDDO-Me effectively enhanced RalBP1 expression to 2.15-fold of control levels in the whole hippocampus following SE (*t*_(12)_ = 5.7, *p* < 0.001 vs. vehicle-treated SE animals, Student *t*-test, *n* = 7; Figure 2A,B and Supplementary Figure S1). Immunofluorescent studies revealed that CDDO-Me elevated RalBP1 expression levels to 2.07 − (*t*_(12)_ = 12.9, *p* < 0.001 vs. vehicle-treated SE animals, Student *t*-test, *n* = 7) and 1.29-fold (*t*_(12)_ = 7.6, *p* < 0.001 vs. vehicle-treated SE animals, Student *t*-test, *n* = 7) of vehicle-treated animal levels in the CA1 region and the dentate gyrus, respectively (Figure 2C,D). Furthermore, CDDO-Me reduced 4-HNE signals to 0.51 − (*t*_(12)_ = 12.5, *p* < 0.001 vs. vehicle-treated SE animals, Student *t*-test, *n* = 7) and 0.75-fold (*t*_(12)_ = 7.5, *p* < 0.001 vs. vehicle-treated SE animals, Student *t*-test, *n* = 7) of vehicle-treated animal levels in the CA1 region and the dentate gyrus, respectively (Figure 2C,E). CDDO-Me also attenuated SE-induced CA1 neuronal degeneration (*t*_(12)_ = 9.5, *p* < 0.001 vs. vehicle-treated SE animals, Student *t*-test, *n* = 7; Figure 2C,F). These findings indicate that RalBP1 expression may be inversely relevant to 4-HNE following SE, and that CDDO-Me may increase CA1 neuronal viability by enhancing RalBP1-mediated 4-HNE efflux following SE.

### 3.2. CDDO-Me Facilitates Mitochondrial Fission in CA1 Neurons under Physiological and Post-SE Conditions

Since the dysregulation of mitochondrial dynamics leads to programmed necrosis in CA1 neurons following SE [8,9,18], we validated whether CDDO-Me affects mitochondrial dynamics in CA1 neurons under physiological and post-SE conditions.

**Figure 3:**
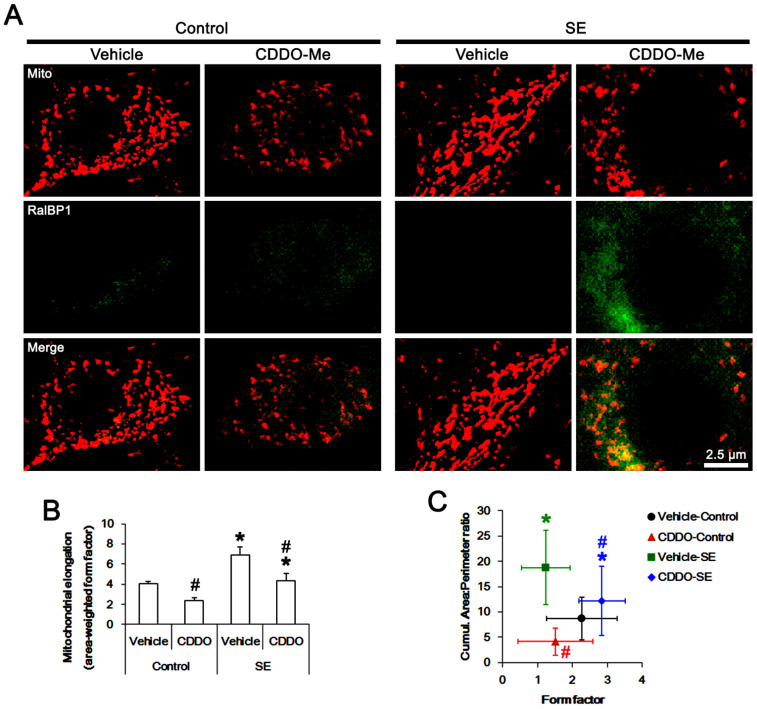
Effects of CDDO-Me on mitochondrial dynamics and RalBP1 expression following SE. As compared to vehicle, CDDO-Me facilitates mitochondrial fission in CA1 neurons under physiological and post-SE conditions. (**A**) Representative photos of mitochondria (Mito) and RalBP1 expression in CA1 neurons. (**B**) Quantification of mitochondrial elongation following SE (mean ± S.E.M.; *,^#^ *p* < 0.05 vs. control animals and vehicle; *n* = total 25 cells in each group; one-way ANOVA). (**C**) Quantification of the cumulative area/perimeter ratio and the form factor following SE (mean ± S.E.M.; *,^#^ *p* < 0.05 vs. control animals and vehicle; *n* = total 25 cells in each group; one-way ANOVA).

Under physiological conditions, CDDO-Me decreased area-weighted form factor (indicating mitochondrial length [19,20]) to 0.59-fold of vehicle levels in CA1 neurons (*F*_(1,48)_ = 8.3, *p* = 0.006 vs. vehicle, one-way ANOVA; Figure 3A,B). CDDO-Me also decreased to 0.48-fold of vehicle-treated animal levels (*F*_(1,48)_ = 7.1, *p* = 0.01 vs. vehicle, one-way ANOVA) and the form factor to 0.66-fold of vehicle-treated animal levels (*F*_(1,48)_ = 6.28, *p* = 0.016 vs. vehicle, one-way ANOVA; Figure 3A,C). Regarding the unaltered RalBP1 expression levels induced by CDDO-Me (Figure 2A,B), these findings indicate that CDDO-Me may accelerate mitochondrial fission in CA1 neurons under physiological conditions, independently of RalBP1 levels.

Consistent with our previous study [9], SE resulted in a 1.73-fold increase in mitochondrial length (*F*_(1,48)_ = 11.9, *p* = 0.001 vs. vehicle-treated control animals, one-way ANOVA; Figure 3A,B). SE also increased the cumulative area/perimeter ratio to 2.16-fold of control animal levels (*F*_(1,48)_ = 28.6, *p* < 0.001 vs. vehicle-treated control animals, one-way ANOVA), while it reduced the form factor to 0.54-fold of control animal levels (*F*_(1,48)_ = 17.7, *p* < 0.001 vs. vehicle-treated control animals, one-way ANOVA; Figure 3A,C). These findings indicate that SE may lead to aberrant mitochondrial fusions and their aggregations in CA1 neurons. As compared to vehicle, CDDO-Me attenuated the SE-induced increases in mitochondrial length to 0.63-fold of vehicle-treated SE animal levels (*F*_(1,48)_ = 8.5, *p* = 0.005 vs. vehicle-treated SE animal levels, one-way ANOVA; Figure 3A,B), accompanied by RalBP1 upregulation (Figure 2 and Figure 3A). However, CDDO-Me also reduced the cumulative area/perimeter ratio to 0.65-fold of vehicle-treated animal levels (*F*_(1,48)_ = 6.98, *p* = 0.01 vs. vehicle, one-way ANOVA) but increased the form factor to 2.3-fold of vehicle-treated animal levels (*F*_(1,48)_ = 7.6, *p* = 0.008 vs. vehicle, one-way ANOVA) following SE (Figure 3A,C). Taken together, our findings suggest that CDDO-Me-induced RalBP1 upregulation may abrogate the abnormal mitochondrial elongations induced by SE.

### 3.3. RalBP1 siRNA Does Not Affect Mitochondrial Dynamics and CA1 Neuronal Death Induced by SE

**Figure 4:**
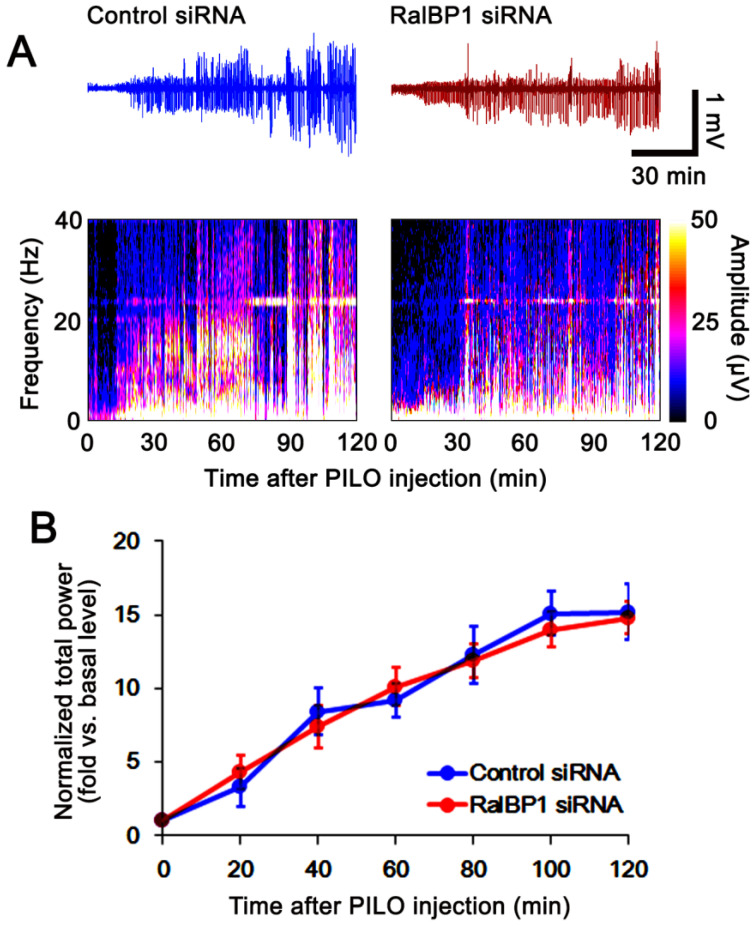
Effects of control siRNA and RalBP1 siRNA on pilocarpine (PILO)-induced seizure susceptibility. RalBP1 knockdown does not influence seizure susceptibility to pilocarpine. (**A**) Representative EEG traces and frequency/amplitude maps following pilocarpine treatment. (**B**) Quantification of the total EEG power (seizure intensity) following pilocarpine injection (mean ± S.E.M.; *n* = 7; repeated-measures ANOVA).

**Figure 5:**
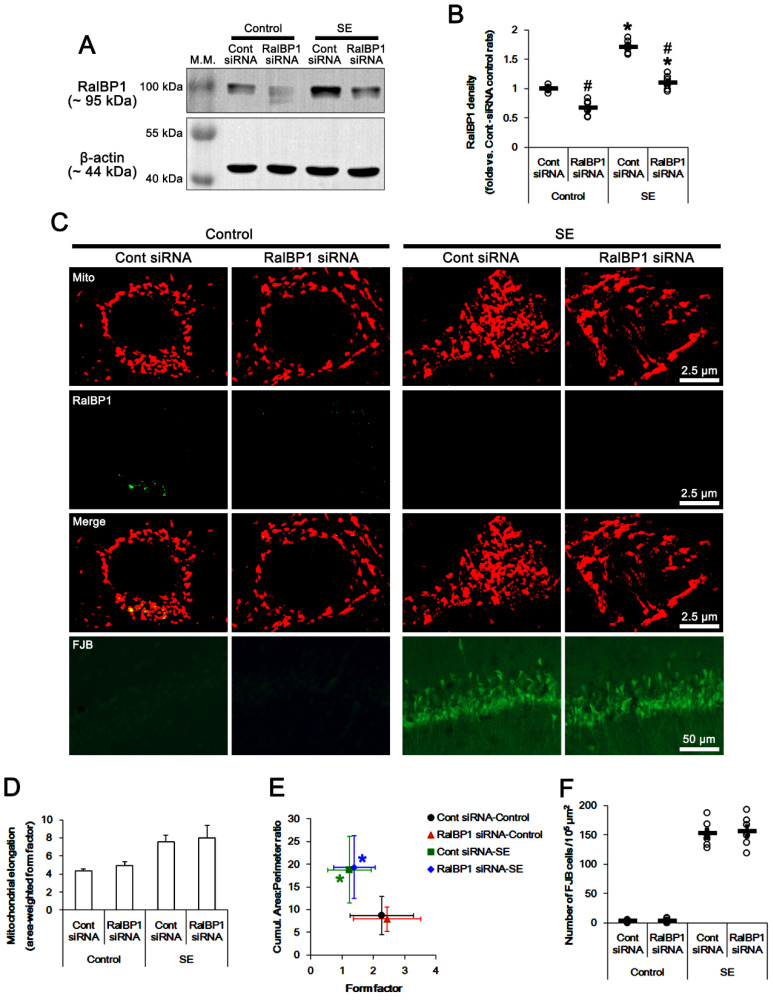
Effects of RalBP1 knockdown on mitochondrial dynamics following SE. RalBP1 siRNA reduces RalBP1 protein levels under physiological and post-SE conditions. However, RalBP1 siRNA does not affect mitochondrial dynamics but aggravates SE-induced CA1 neuronal death. (**A**) Representative Western blots of RalBP1 protein levels in the whole hippocampus (M.W. marker, molecular weight marker). (**B**) Quantification of RalBP1 protein levels based on Western blot data. Open circles indicate each individual value. Horizontal bars indicate mean value (mean ± S.E.M.; *,^#^ *p* < 0.05 vs. control animals and control siRNA; *n* = 7; one-way ANOVA). (**C**) Representative photos of mitochondria (Mito) and RalBP1 expression in CA1 neurons. (**D**) Quantification of mitochondrial elongation index following SE (mean ± S.E.M.). (**E**) Quantification of the cumulative area/perimeter ratio and the form factor following SE (mean ± S.E.M.). (**F**) Quantification of the number of FJB-positive CA1 neurons following SE (mean ± S.D.). Open circles indicate each individual value. Horizontal bars indicate mean value (*n* = 7).

To elucidate the role of RalBP1 in SE-induced aberrant mitochondrial dynamics in CA1 neurons, we applied RalBP1 knockdown prior to SE induction. As compared to control siRNA, RalBP1 siRNA did not influence the seizure susceptibility to pilocarpine (repeated one-way ANOVA; *n* = 7; Figure 4). Under physiological conditions, RalBP1 siRNA reduced RalBP1 protein levels 0.67-fold of control siRNA levels in the whole hippocampus (*t*_(12)_ = 6.6, *p* < 0.001 vs. control siRNA-treated control animals, Student *t*-test, *n* = 7; Figure 5A,B and Supplementary Figure S1). RalBP1 knockdown also did not affect mitochondrial dynamics under physiological conditions (Figure 5C–E). Furthermore, RalBP1 siRNA did not influence the aberrant mitochondrial fusion and CA1 neuronal loss induced by SE (Figure 5C–F), although it decreased RalBP1 protein levels 0.64-fold of control siRNA levels in the whole hippocampus (*t*_(12)_ = 6.6, *p* < 0.001 vs. control siRNA-treated control animals, Student *t*-test, *n* = 7; Figure 5A,B). These findings indicate that endogenous RalBP1 may not be involved in mitochondrial dynamics under physiological conditions. Considering the effects of CDDO-Me on mitochondrial dynamics and RalBP1 expression following SE, however, it is likely that SE-induced RalBP1 downregulation may lead to aberrant mitochondrial elongations in CA1 neurons. To confirm this, we applied cotreatment of RalBP1 siRNA with CDDO-Me prior to SE induction.

### 3.4. RalBP1 Knockdown Diminishes the Effects of CDDO-Me on Aberrant Mitochondrial Dynamics and CA1 Neuronal Death Following SE

**Figure 6:**
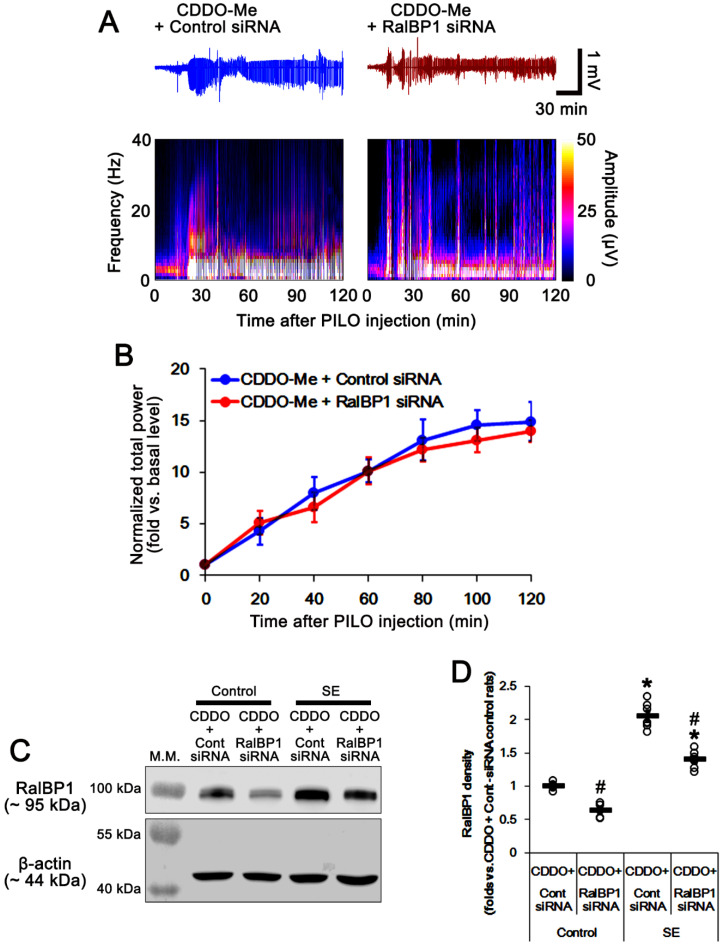
Effects of cotreatment of RalBP1 siRNA with CDDO-Me (CDDO) on pilocarpine (PILO)-induced seizure activity. RalBP1 siRNA does not change the seizure susceptibility to pilocarpine. However, RalBP1 siRNA reduces RalBP1 upregulation induced by CDDO-Me under physiological and post-SE conditions. (**A**) Representative EEG traces and frequency/amplitude maps following pilocarpine injection. (**B**) Quantification of the total EEG power (seizure intensity) in response to pilocarpine (mean ± S.E.M.; *n* = 7; repeated-measures ANOVA). (**C**) Representative Western blots of RalBP1 protein levels in the whole hippocampus (M.W. marker, molecular weight marker). (**D**) Quantification of RalBP1 protein levels based on Western blot data. Open circles indicate each individual value. Horizontal bars indicate mean value (mean ± S.E.M.; *,^#^ *p* < 0.05 vs. control animals and control siRNA; *n* = 7; one-way ANOVA).

In the present study, cotreatment of RalBP1 siRNA with CDDO-Me did not change the seizure susceptibility to pilocarpine, as compared to cotreatment of control siRNA with CDDO-Me (repeated one-way ANOVA; *n* = 7; Figure 6A,B). Under physiological conditions, RalBP1 siRNA cotreatment reduced RalBP1 protein levels 0.63-fold of control siRNA levels in the whole hippocampus (*t*_(12)_ = 9.9, *p* < 0.001 vs. control siRNA cotreatment, Student *t*-test, *n* = 7; Figure 6C,D and Supplementary Figure S1). Following SE, RalBP1 siRNA cotreatment also decreased RalBP1 protein levels 0.68-fold of control siRNA levels in the whole hippocampus (*t*_(12)_ = 7.4, *p* < 0.001 vs. control siRNA cotreatment, Student *t*-test, *n* = 7; Figure 6C,D and Supplementary Figure S1). These findings indicate that RalBP1 knockdown may effectively diminish CDDO-Me-induced RalBP1 upregulation following SE.

**Figure 7:**
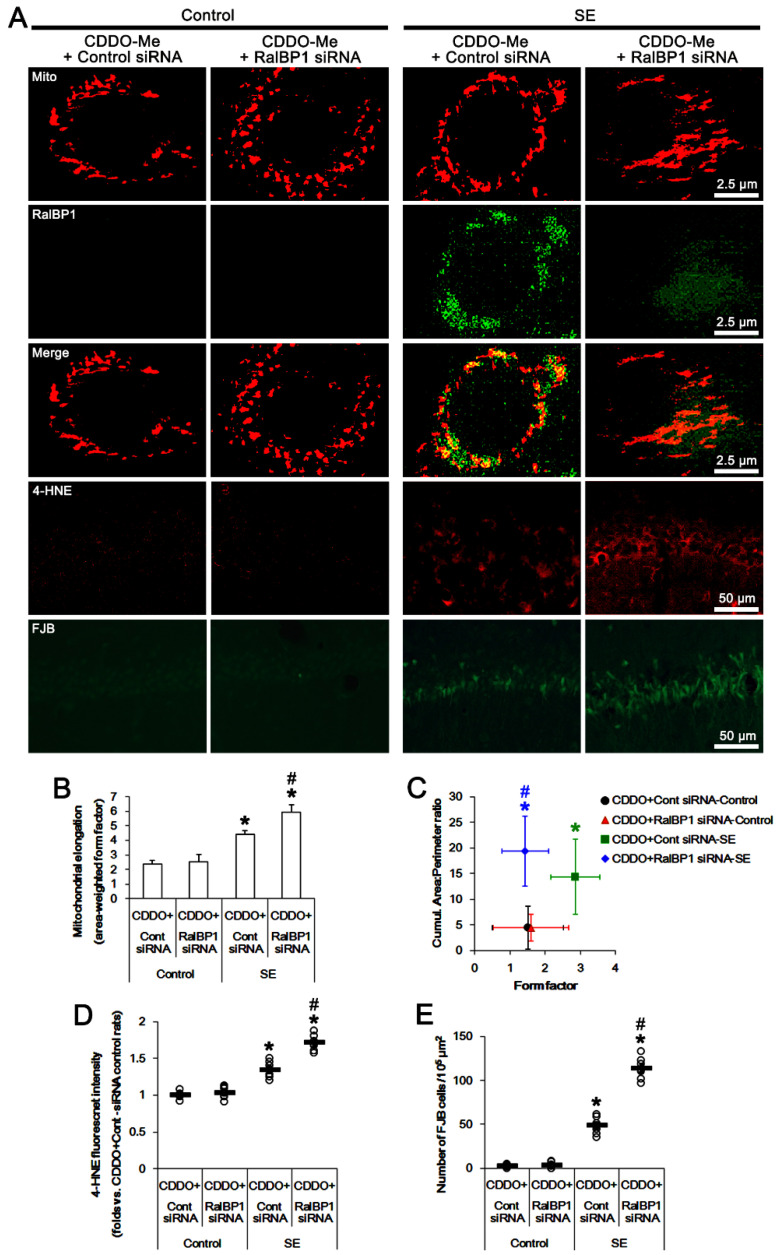
Effects of cotreatment of RalBP1 siRNA on CDDO-Me-induced mitochondrial fission and RalBP1 expression following SE. RalBP1 siRNA cotreatment abrogates the effect of CDDO-Me on mitochondrial fission, 4-HNE accumulation, and neuronal death in CA1 neurons following SE. (**A**) Representative photos of mitochondria (Mito), RalBP1 expression, 4-HNE accumulation, and SE-induced neuronal damage in CA1 neurons. (**B**) Quantification of mitochondrial elongation index following SE (mean ± S.E.M.; *,^#^ *p* < 0.05 vs. control animals and control siRNA; *n* = total 25 cells in each group; one-way ANOVA). (**C**) Quantification of the cumulative area/perimeter ratio and the form factor following SE (mean ± S.E.M.; *,^#^ *p* < 0.05 vs. control animals and control siRNA; *n* = total 25 cells in each group; one-way ANOVA). (**D**) Quantification of 4-HNE fluorescent intensity (mean ± S.E.M.; *,^#^ *p* < 0.05 vs. control animals and control siRNA; *n* = 7; Student *t*-test). (**E**) Quantification of the number of FJB-positive CA1 neurons following SE (mean ± S.D.; *,^#^ *p* < 0.05 vs. control animals and control siRNA; *n* = 7; Student *t*-test). Open circles indicate each individual value. Horizontal bars indicate mean value.

Under physiological conditions, RalBP1 siRNA cotreatment did not affect CDDO-Me-induced mitochondrial fragmentations in CA1 neurons (Figure 7A–C). Following SE, however, RalBP1 siRNA cotreatment resulted in 1.35-fold increase in mitochondrial length in CA1 neurons (*F*_(1,48)_ = 7.4, *p* = 0.009 vs. control siRNA cotreatment, one-way ANOVA; Figure 7A,B). RalBP1 siRNA cotreatment also increased the cumulative area/perimeter ratio to 1.35-fold of control siRNA levels (*F*_(1,48)_ = 14.3, *p* < 0.001 vs. control siRNA cotreatment), while it reduced the form factor to 0.5-fold of control siRNA levels (*F*_(1,48)_ = 11.9, *p* = 0.001 vs. control siRNA cotreatment, one-way ANOVA; Figure 7A,C). Furthermore, RalBP1 siRNA cotreatment increased 4-HNE signals to 1.27-fold of control siRNA levels in CA1 neurons (*t*_(12)_ = 24, *p* < 0.001 vs. control siRNA cotreatment, Student *t*-test, *n* = 7), accompanied by reduced RalBP1 protein levels (Figure 7A,D). RalBP1 siRNA cotreatment also aggravated SE-induced CA1 neuronal degeneration (*t*_(12)_ =11, *p* < 0.001 vs. control siRNA cotreatment, Student *t*-test, *n* = 7; Figure 7A,E). Therefore, our findings indicate that CDDO-Me may mitigate SE-induced CA1 neuronal degeneration by facilitating RalBP1-mediated mitochondrial fission and 4-HNE efflux.

## 4. Discussion

The major findings in the present study are that CDDO-ME ameliorated SE-induced aberrant mitochondrial dynamics by RalBP1 upregulation, although RalBP1 alone did not affect mitochondrial dynamics under physiological conditions.

The impaired mitochondrial fission results in mitochondrial dysfunction, including bioenergetics [14,15]. DRP1 S616 phosphorylation plays an important role in mitochondrial fragmentation [3]. Recently, we have reported that CDDO-Me facilitates DRP1-mediated mitochondrial fissions by enhancing its S616 phosphorylation, which attenuates CA1 neuronal death induced by SE [18].

RalBP1 is a multifunctional protein showing transport activity. RalBP1 also fosters DRP1 S616 phosphorylation. Indeed, RalBP1 knockdown reduces the amount of DRP1 S616 phosphorylation and inhibits mitochondrial fragmentation [3]. However, the present data show that RalBP1 knockdown did not alter mitochondrial length in CA1 neurons under physiological conditions and following SE. Considering very low RalBP1 protein levels, these findings indicate that RalBP1 may not participate in mitochondrial fission in CA1 neurons under both conditions. In the present study, SE evoked the defects of mitochondrial fission in CA1 neurons and massive neuronal CA1 neuronal death without altering RalBP1 protein levels. However, CDDO-Me effectively attenuated SE-induced CA1 neuronal death concomitant with reduced mitochondrial length and RalBP1 upregulation, although CDDO-Me did not influence RalBP1 expression levels under physiological conditions. Furthermore, RalBP1 siRNA cotreatment abrogated CDDO-Me-induced mitochondrial fragmentation in CA1 neurons under post-SE conditions. Therefore, these findings suggest that CDDO-Me-induced RalBP1 upregulation may facilitate mitochondrial fission following SE.

RalBP1 also transports the oxidized lipid byproducts, including 4-HNE. Indeed, impaired RalBP1 function cells exert the accumulation of 4-HNE. Therefore, RalBP1 plays an important role in the stress defense mechanism [10,11,21]. In the present study, SE increased RalBP1 expression in dentate granule cells that are invulnerable to SE [22,23]. Furthermore, 4-HNE signals were very low in these cells following SE. However, the present study demonstrates that SE induced 4-HNE signals in CA1 neurons, which was ameliorated by CDDO-Me-induced RalBP1 upregulation. Furthermore, RalBP1 siRNA cotreatment diminished the effect of CDDO-Me on 4-HNE induction and CA1 neuronal death following SE. These findings indicate that RalBP1 expression may be inversely relevant to SE-induced 4-HNE synthesis, and that CDDO-Me may also increase CA1 neuronal viability by enhancing RalBP1-mediated 4-HNE efflux following SE. Therefore, SE-induced RALBP1 upregulation in dentate granule cells may be involved in 4-HNE efflux as an adaptive response to oxidative stress. Considering the antioxidant properties of CDDO-Me [16,17], it is postulated that CDDO-Me would attenuate abnormal mitochondrial elongation by reducing oxidative stress following SE. If it is true, CDDO-Me would abrogate SE-induced RalBP1 upregulation in dentate granule cells that are resistant to SE insults [22,23]. However, the present study shows that CDDO-Me greatly increased RalBP1 protein levels in dentate granule cells under post-SE conditions. Therefore, our findings indicate that acceleration of mitochondrial fission induced by CDDO-Me may not be a result of RalBP1-mediated reductions in oxidative stress but a direct result of RalBP1 upregulation.

RalBP1 knockout mice show lower blood glucose levels than wild-type mice [24], which also triggers mitochondrial fission in CA1 neurons [25]. However, the present data show that RalBP1 siRNA cotreatment alleviated mitochondrial fission induced by CDDO-Me following SE, although RalBP1 siRNA alone could not affect mitochondrial dynamic under physiological conditions and following SE. Therefore, it is plausible that CDDO-Me-induced RalBP1 upregulation may facilitate mitochondrial fragmentations, independent of glucose metabolism.

Although the underlying mechanisms of CDDO-Me-induced RalBP1 upregulation have still been unclear, it is considerable that nuclear factor-κB (NF-κB) and p300 transactivate RalBP1 expression [26,27]. RalBP1 is a target of proinflammatory molecules (such as tumor-necrosis factor-α) via a cluster of evolutionarily conserved NF-κB binding sites [27]. However, it is well known that CDDO-Me inhibits NF-κB-mediated signaling pathways [28,29,30,31]. Therefore, it is unlikely that CDDO-Me would increase RalBP1 expression by activating the NF-κB signaling pathway. On the other hand, p300 knockdown decreases RalBP1 expression, indicating that p300 regulates RalBP1 promoter activity and its expression [26]. Interestingly, p300 promotes Nrf2 transcriptional activity, thereby promoting Nrf2 nuclear localization, since p300 competes with Kelch-like erythroid cell-derived protein with CNC homology-associated protein 1 (Keap1), a repressor of Nrf2, for NRF2 binding [32]. Thus, it is likely that p300 may act as an endogenous Nrf2 activator. Indeed, p300 overexpression increases cell viability against oxidative stress [32]. Similarly, CDDO-Me exerts the release of Nrf2 from Keap1, which hinders its ubiquitination and increases Nrf2 activity [33]. Under these conditions, CDDO-Me may provide an opportunity to increase the binding of p300 to RalBP1 promoter sites. Therefore, it is likely that CDDO-Me may increase RalBP1 protein expression by enhancing p300 transcriptional activity under pathophysiological conditions. Further studies concerning the effect of CDDO-Me on p300-mediated RalBP1 transactivation are needed in the near future.

On the other hand, the present data demonstrating the mitochondrial hyperfusion-mediated CA1 neuronal death under post-SE conditions are opposite to those under other pathophysiological conditions. Unlike other neurological diseases, including Alzheimer’s disease, cerebrovascular disease (stroke), Huntington’s disease, and Parkinson’s disease [2], seizure activity leads to dysregulation of mitochondrial fission coupled with impairments of their distribution and function [34,35]. Indeed, *DRP1* pathogenic variant-mediated mitochondrial elongation is relevant to seizures in human patients [36]. In particular, fibroblasts obtained from refractory epilepsy patients show the defects of DRP1-mediated mitochondrial fission without altering bioenergetics [37]. In previous studies [8,38], we have also reported that the increased mitochondrial fission induced by WY14643 protects CA1 neurons from SE, whereas the mitochondrial elongation by Mdivi-1 exacerbates SE-induced CA1 neuronal death. Therefore, aberrant mitochondrial fusion may be involved in the pathogenesis of SE-induced neuronal death.

## 5. Limitation of the Study

Lucchi et al. [39] reported that pilocarpine-induced SE leads to neuronal degenerations, including hilus interneurons and entorhinal cortical neurons concomitant with CA1 neurons. In previous studies [29,38,40,41,42], we have also reported that pilocarpine-induced SE develops neuronal damage in the hilus of the hippocampus, entorhinal cortex, and piriform cortex. Unlike CA1 neurons, however, CDDO-Me does not protect hilus interneurons, although it decreases 4-HNE levels in these neurons following SE [18]. Therefore, SE-induced hilus neuronal death is irrelevant to RalBP1-mediated 4-HNE efflux. Indeed, we have revealed that SE results in hilus neuronal damage by excessive mitochondrial fragmentation through the p47Phox/cyclin-dependent kinase 5/dynamin-related protein 1 signaling pathway [43] and mitochondrial translocation of caspase-3 [38], which are not involved in SE-induced CA1 neuronal death. Furthermore, SE induces severe vasogenic edema in the entorhinal and piriform cortices, which is limited in the hippocampus [42]. Since SE-induced vasogenic edema formations in these regions are prior to neuronal degeneration and the prevention of vasogenic edema attenuates neuronal death in these regions [44,45], vasogenic edema may be one of the underlying mechanisms in the neurodegeneration in the entorhinal and piriform cortices. CDDO-Me also ameliorates neuronal death in the piriform and entorhinal cortices and increases astroglial viability by inhibiting NF-κB-mediated vasogenic edema formation [29]. In addition, 4-HNE upregulation is restricted to astrocytes concomitant with vasogenic edema formation in the piriform cortex [46]. Therefore, it is likely that CDDO-Me may mitigate SE-induced neuronal degeneration in the entorhinal and piriform cortices by inhibiting vasogenic edema formation, but not activating RalBP1-mediated 4-HNE efflux from cortical neurons. For these reasons, we focused on CA1 neurons to evaluate the effects of CDDO-Me and RalBP1 siRNA on 4-HNE levels during SE-induced neuronal death in the present study and suggest that the underlying mechanisms of SE-neuronal degeneration may be complicated and heterogenous.

On the other hand, Lucchi et al. [39] applied diazepam treatment 10 min after the pilocarpine-SE onset to improve survival and to standardize SE duration based on the paper of Gualtieri et al. [47]. The early diazepam administration rapidly reduces motor seizure severity (tonic seizures) without altering subtle convulsive movements, such as clonic jerks, whiskers or eyelids, and tremors of the whole body. In addition, this early diazepam treatment dampens, but does not abolish, electrocorticographic seizure activity [47]. In the present study, we applied a 2 h lasting SE rat model. In this model, diazepam treatment at 2 h after SE onset effectively decreases pilocarpine-induced seizure activities on hippocampal EEG, as well as behavioral seizures [48,49]. Therefore, our model provides a long observation to evaluate the anticonvulsive effects of test chemicals without the interference effect of diazepam, as compared to the model of Lucchi et al. [39]. Most of all, Gualtieri et al. [47] reported that the results obtained from the early diazepam-treated model are consistent with those of our 2 h lasting SE model, citing our papers [40,42,44]. As compared to vehicle and control siRNA, the present study shows that CDDO-Me and RalBP siRNA did not affect the seizure latency and EEG power (seizure severity) induced by pilocarpine during 2 h EEG recording. Considering that EEG recording is more reliable to quantify the seizure onset and its severity than the observation of behavioral seizures, our results indicate that CDDO-Me or RalBP siRNA may not have anticonvulsive properties and may regulate CA1 neuronal death without altering pilocarpine-induced SE activity. Further studies are needed to validate the efficacies of diazepam treatment at different time points after SE onset for evaluating anticonvulsants.

## 6. Conclusions

In the present study, we found that CDDO-Me upregulated RalBP1 expression, accompanied by a decrease in 4-HNE signals in CA1 neurons following SE. CDDO-Me reduced mitochondrial length in these neurons under physiological and post-SE conditions. Although RalBP1 knockdown alone did not affect mitochondrial dynamics and CA1 neuronal death induced by SE, RalBP1 siRNA cotreatment abrogated the effects of CDDO-Me on aberrant mitochondrial dynamics and CA1 neuronal death following SE. Therefore, our findings indicate that CDDO-Me may ameliorate CA1 neuronal degeneration by accelerating RalBP1-mediated mitochondrial fission and 4-HNE efflux following SE.

## Figures and Tables

**Table 1:** Primary antibodies used in the present study.

Antigen	Host	Manufacturer (Catalog Number)	Dilution Used
4-HNE	Rabbit	Alpha Diagnostic (#HNE11-S)	1:500 (IH)
Mitochondrial marker (Mitochondrial complex IV subunit 1, MTCO1)	Mouse	Abcam (#ab14705)	1:500 (IH)
RalBP1	Rabbit	Abcam (#ab133549)	1:500 (IH) 1:10,000 (WB)
β-actin	Mouse	Sigma (#A5316)	1:6000 (WB)

IH: immunohistochemistry; WB, Western blot.

## Data Availability

The data are contained within the article and Supplementary Materials.

## Supplementary Materials

The following supporting information can be downloaded at: https://www.mdpi.com/article/10.3390/antiox11050985/s1, Figure S1: Full-length gel images of Western blot data in Figure 2A, Figure 5A and Figure 6C.
