# Supplementary material for: CDDO-Me Attenuates CA1 Neuronal Death by Facilitating RalBP1-Mediated Mitochondrial Fission and 4-HNE Efflux in the Rat Hippocampus Following Status Epilepticus
Source: Antioxidants (Basel). 2022 May 18;11(5):985. doi: 10.3390/antiox11050985 (PMC9137584; doi:10.3390/antiox11050985)
Supplement: Supplementary file 1 [file antioxidants-11-00985-s001.zip › antioxidants-1671246-supplementary.pdf]

Supplementary information

CDDO-Me attenuates CA1 neuronal death by facilitating RalBP1-mediated mitochondrial fission and 4-HNE efflux in the rat hippocampus following status epilepticus

Ji-Eun Kim [1,*], Duk-Shin Lee [1] , Tae-Hyun Kim [1] and Tae-Cheon Kang [1,*]

[1] Department of Anatomy and Neurobiology and Institute of Epilepsy Research, College of Medicine, Hallym University, Chuncheon 24252, Korea; dslee84@hallym.ac.kr (D-SL), hyun1028@hallym.ac.kr (T.-H.K.).

***** Correspondence: jieunkim@hallym.ac.kr; Tel: +82-33-248-2524; Fax: +82-33-248-2525 and tckang@hallym.ac.kr; Tel: +82-33-248-2524; Fax: +82-33-248-2525.

Fig. 2

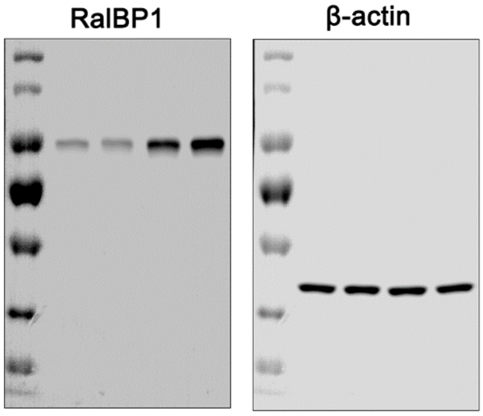

Fig. 5.

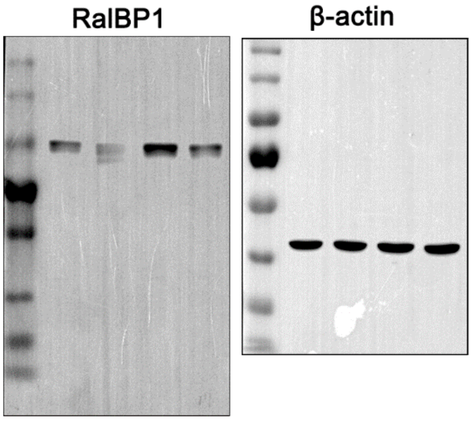

Fig. 6.

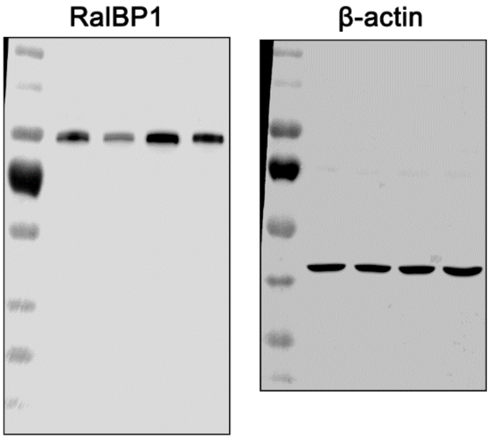

Supplementary Figure S1. Full-length gel images of Western blot data in Figure 2A, 5A and 6C.
